# Supplementary figures and images for: Rhythmicity is linked to expression cost at the protein level but to expression precision at the mRNA level
Source: PLoS Comput Biol. 2022 Sep 12;18(9):e1010399. doi: 10.1371/journal.pcbi.1010399 (PMC9518874; doi:10.1371/journal.pcbi.1010399)

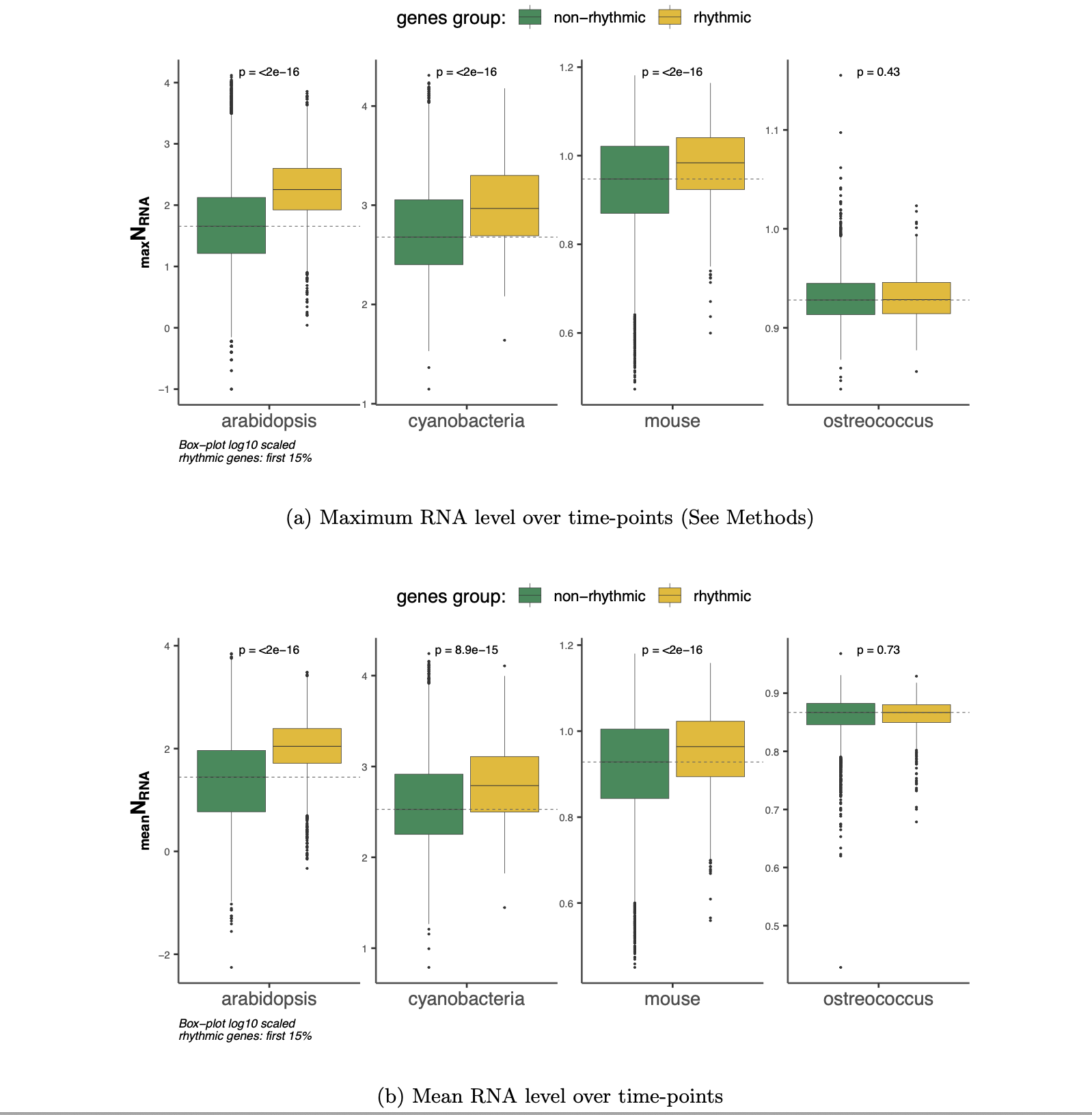

Supplement: S1 Fig — Rhythmic transcripts are highly expressed transcripts. (TIFF) [file pcbi.1010399.s001.tiff]

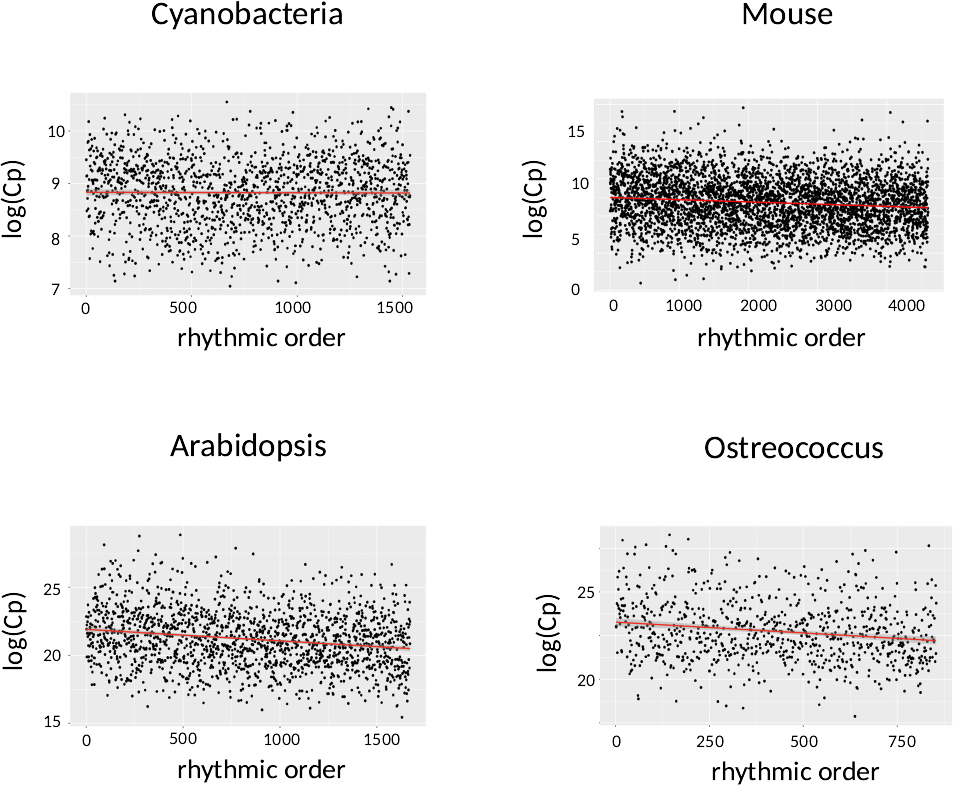

Supplement: S2 Fig — (TIFF) [file pcbi.1010399.s002.tiff]

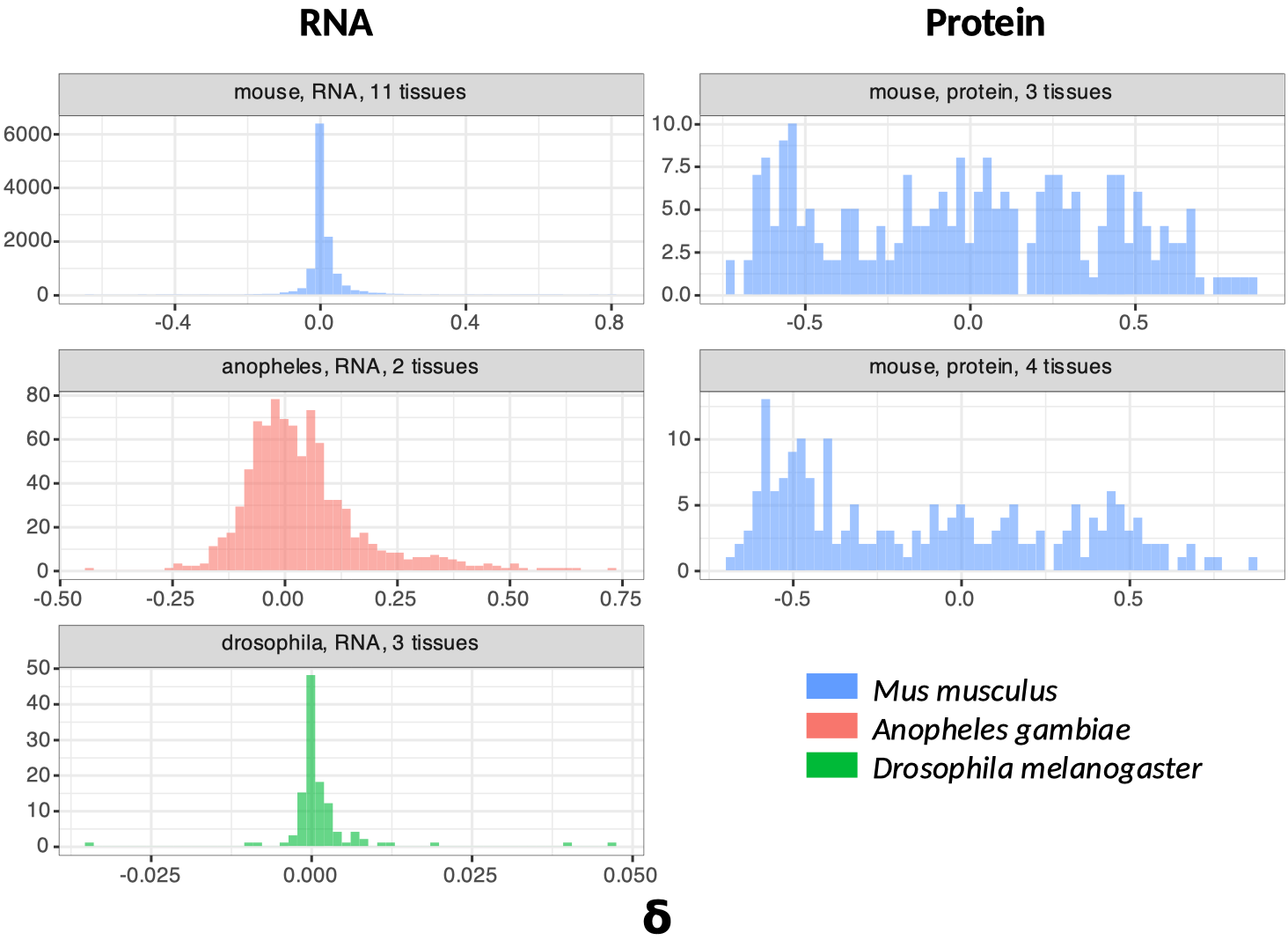

Supplement: S3 Fig — δ shows a bimodal distribution at protein level. (TIFF) [file pcbi.1010399.s003.tiff]

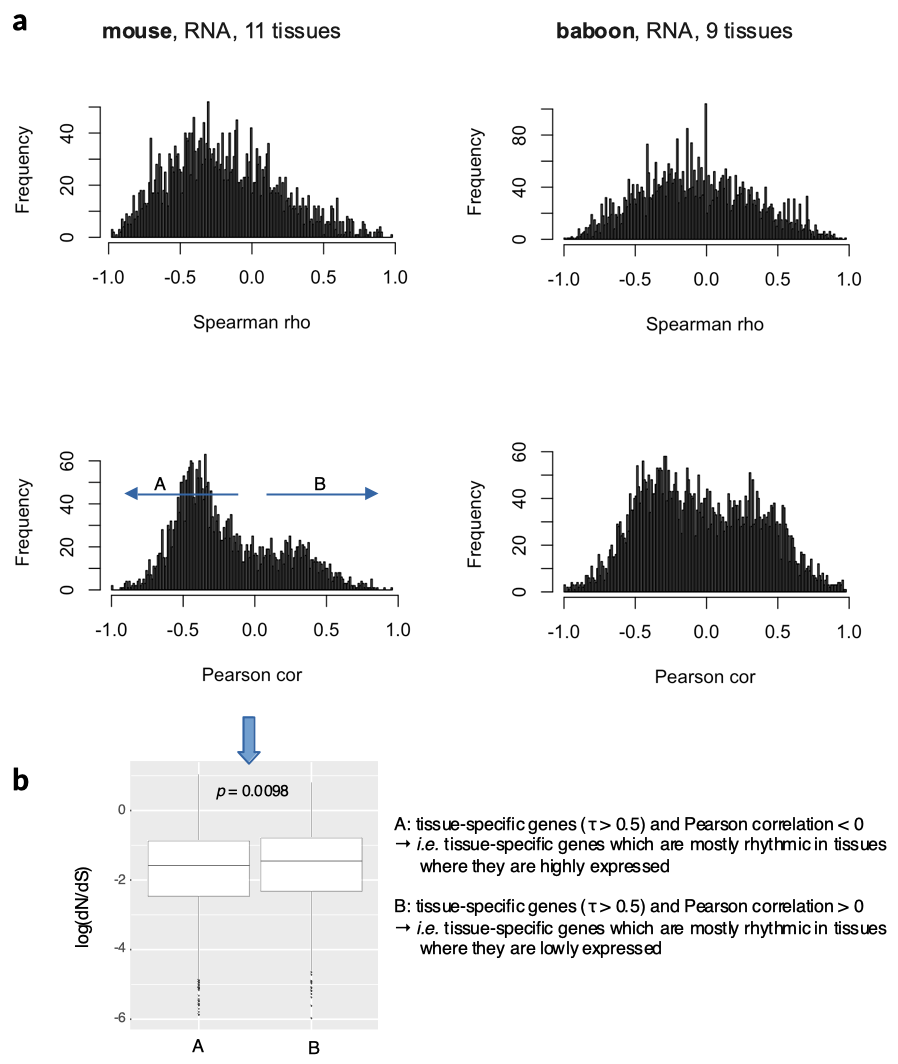

Supplement: S4 Fig — a) Histograms of Pearson’s and Spearman’s coefficients testing the correlation between the expression level and the rhythmicicity signal (rhythm p-value) measured across the body for every tissue-specific genes (τ >0.5). b) Student’s t-test comparing the mean of dN/dS between gene sets A and B: Tissue-specific genes which are mostly rhythmic in tissues where they are highly expressed are under stronger selective constraint than those which are rhythmic in tissues where they are lowly expressed. (TIFF) [file pcbi.1010399.s004.tiff]

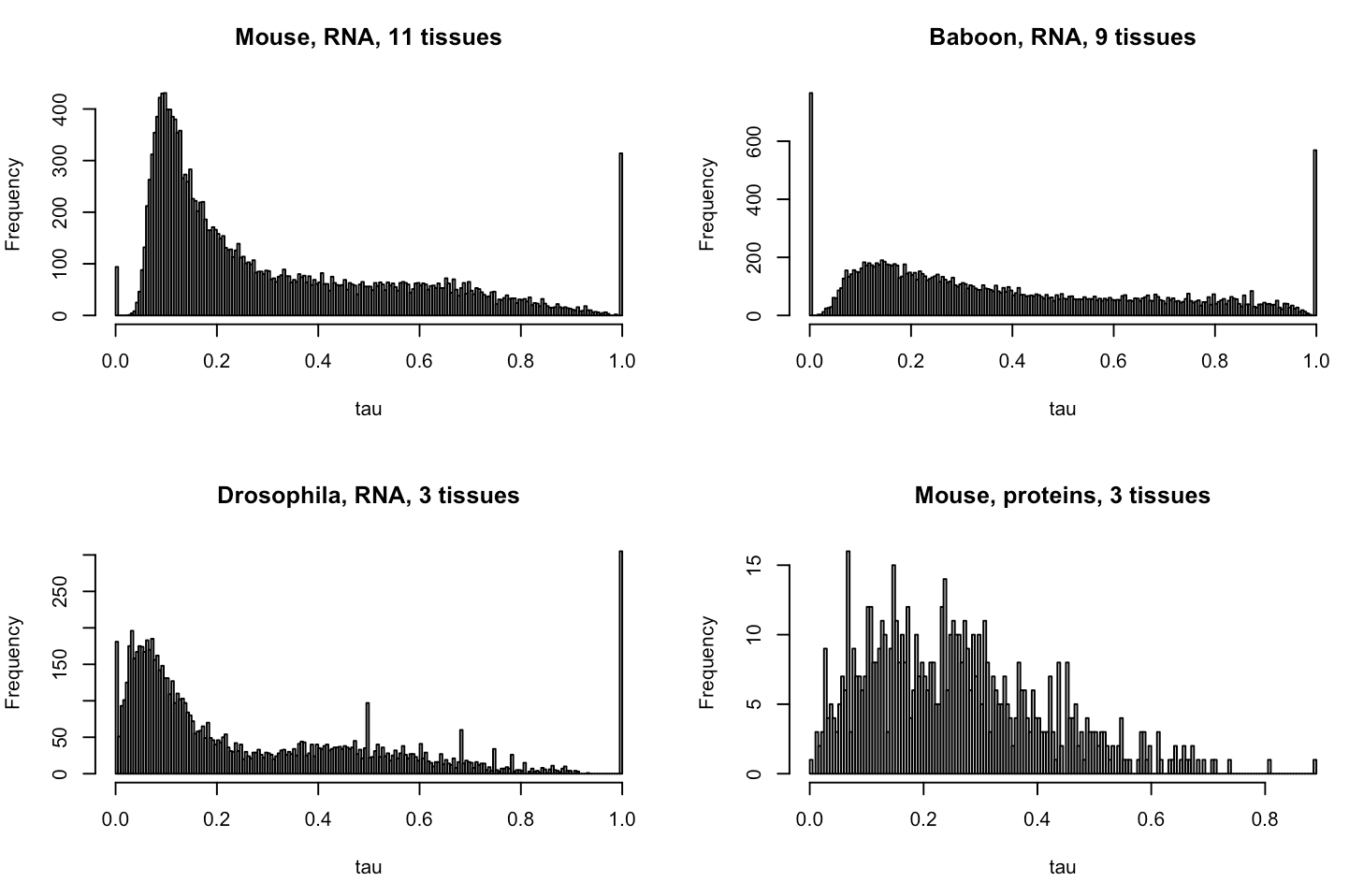

Supplement: S7 Fig — (TIFF) [file pcbi.1010399.s007.tiff]
