# Supplementary material for: Rhythmicity is linked to expression cost at the protein level but to expression precision at the mRNA level
Source: PLoS Comput Biol. 2022 Sep 12;18(9):e1010399. doi: 10.1371/journal.pcbi.1010399 (PMC9518874; doi:10.1371/journal.pcbi.1010399)
Supplement: Attachment — Submitted filename: LaloumPlosCompBioReview.pdf [file pcbi.1010399.s018.pdf]

Review of “Rhythmicity is linked to expression cost at the protein level but to expression precision at the mRNA level”

In this work, the authors explore the question of why many genes and proteins have rhythmic expression. They give an evolutionary explanation that rhythmic protein expression minimizes costs, while rhythmic RNA levels reduce expression noise. They also find that genes with high purifying selection are also rhythmically expressed, supporting the theory that the role of rhythms is to reduce expression noise. They further argue that the tissue-specific nature of the rhythms also supports this theory.

The authors present a novel approach to considering the basis of rhythmicity in genes, based on biological costs. While the authors offer much supporting evidence for their theory, their arguments would be more convincing with more consideration of other possible explanations (particularly the need to anticipate timing and toxicity effects). Additional experiments to help verify their claim would also strengthen their argument as this analysis cannot show causality. Their response to previous reviewers have greatly improved the arguments in the paper, and below, I offer some additional points to address to further clarify the arguments as well as some minor changes to aid the reader.

Major Comments:

Cyclicality of costliest genes

- Has formula 1 been verified or is it based on assumptions that the cost should be linear in length and number? Number seems sensible to be linear, but length seems that other forms may be reasonable. Further, how different is this result when considering the individual amino acids that make up a protein rather than their average cost, particularly given that these amino acids have different lengths (relatedly is the length of the protein captured in the cost of the amino acid synthesis)?

Discussion

- In a number of places, the authors refer to the cost of temporal regulation. This is not quantified by any formulae in the paper. How does this compare to the cost of protein expression? Particularly given the notes here that these calculated costs are based on a steady state.
- Do the different phases of genes and proteins make it so that costs are relatively constant across the 24-cycle? Many genes and proteins are active at dusk and dawn – is there an evolutionary hypothesis for these times which would seemingly have concentrated periods of high energy cost?

Questions for Consideration:

Introduction

- The authors argue that the fact that some genes are constant or absence in some tissues but cycle in others is evidence that rhythmic regulation has costs. They claim this is due to the temporal precision required, but is it actually just costly in the amount of gene/protein produced? In other words, do the tissues with constant levels of the gene/protein produce less over the course of 24 h, and hence the higher cost is actually just more production (rather than the timing specific regulations)?

- Are additional mechanisms required to prevent rhythmicity at the protein level when RNA is rhythmic? How do the costs of preventing rhythmicity in these cases compare to the costs of rhythmicity?

Cyclicality of costliest genes

- It seems to me that the discussion of greater costs for higher expression levels of proteins is central to the authors' arguments and should be moved to the main text?
- It may be valuable to consider a 3 dimensional plot of expression level vs. length vs. cost to better show that the cost is being driven by expression more than length.

Genes tend to be rhythmic in the tissues in which they are highly expressed

- How are the expression levels determined? Do you compare total expression, mean expression, or expression at the peak? Have the p-values been appropriately adjusted for multiple comparisons?
- It would help to at least refer to the methods section here for a definition of tau.

Lower cell-to-cell variability of genes with rhythmic transcripts

- How is the timepoint at which noise is assessed chosen? There are a number of claims here that are not particularly clear as to why this is generalized?
- Relatedly, in comparing noise – are differences in expression do to differences in amplitude, phase, or both?

Discussion

- Have you considered genes/proteins with ultradian rhythms? Or is there something unique to the circadian length cycle that results in evolutionary advantage? Is there a difference in genes/proteins that can be classified as circadian (endogenous) and others? How many of the genes/proteins are downstream of the core circadian clock and does that explain the rhythmicity?
- Are there additional experiments that could be proposed to test your theory? For example, thinking about the toxicity of proteins that oscillate or that levels are needed for protein-protein interactions? Or ways to manipulate RNA expression to more directly test for noise effects?

Materials and Methods

- How do you account for genes/proteins that have a period of near 24 h but not exactly 24 h? Do you renormalize to make comparisons?

Minor Comments and Questions:

Introduction

- I don't understand what the clause "translation is the major contribution to making expression costs visible to selection" means. Why are some processes "invisible" to selection? What does it mean to be "visible"? Do you mean easily quantifiable?

Figure 1b

- I don't understand the connection between the middle and right panels. The middle shows a single protein of each class, and the right shows a box plot – where does the variation in the box plot come from? How is daily contribution to fitness calculated and where does the cost (very clearly illustrated in the middle panel) factor in?

Figure 1c

- It would be helpful to overlay a narrow and flat fitness function to show the relationship between the two curves – this makes intuitive sense but showing the match between the

peak protein expression and the shape of the fitness function would make this relationship more explicit.

Genes tend to be rhythmic in the tissues in which they are highly expressed

- Please include which test is being used for rhythmicity with the p-value.
- I understand the motivation for ignoring intermediate p-values, but how did you choose the p-value cutoffs? Is the significance of your results sensitive to the chosen cutoff?

Minor Edits and Corrections:

- Cyclicality of costliest genes
- Move equation 1 to appear before "other costs such as AA polymerization …" to be easier for the reader so that the notation and formula appear close together

Figure 1

- Consider moving the panel labeling to appear at the upper left rather than lower left. For example, in reading the caption, it was hard to tell whether what is supposed to be panel b was part of panel a.

Discussion

- "but as *be* a by-product of the selection" – remove this extra word typo

Materials and Methods

- Title of this section should be Materials *AND* Methods
- In papio anubi, "time-serie**s**" – add the "s"
