# Supplementary material for: Rhythmicity is linked to expression cost at the protein level but to expression precision at the mRNA level
Source: PLoS Comput Biol. 2022 Sep 12;18(9):e1010399. doi: 10.1371/journal.pcbi.1010399 (PMC9518874; doi:10.1371/journal.pcbi.1010399)
Supplement: Attachment — Submitted filename: Revision.pdf [file pcbi.1010399.s020.pdf]

- **General Statements**

We thank reviewers for helping us clarify our manuscript. Some key information was only in Supporting Information document, and was not obvious to find. We have now introduced some of this information into the main text, and otherwise clarified to which specific sub-paragraph of the Supporting Information document we refer every time we mention it.

- **Point-by-point description of the revisions**

Purple Italics: old text

Black Italics: new text

REVIEWER 1

In this work, the authors explore the question of why many genes and proteins have rhythmic expression. They give an evolutionary explanation that rhythmic protein expression minimizes costs, while rhythmic RNA levels reduce expression noise. They also find that genes with high purifying selection are also rhythmically expressed, supporting the theory that the role of rhythms is to reduce expression noise. They further argue that the tissue-specific nature of the rhythms also supports this theory.

The authors present a novel approach to considering the basis of rhythmicity in genes, based on biological costs. While the authors offer much supporting evidence for their theory, their arguments would be more convincing with more consideration of other possible explanations (particularly the need to anticipate timing and toxicity effects). Additional experiments to help verify their claim would also strengthen their argument as this analysis cannot show causality. Their response to previous reviewers have greatly improved the arguments in the paper, and below, I offer some additional points to address to further clarify the arguments as well as some minor changes to aid the reader.

Major Comments:

Cyclicality of costliest genes
• Has formula 1 been verified or is it based on assumptions that the cost should be linear in length and number? Number seems sensible to be linear, but length seems that other forms may be reasonable. Further, how different is this result when considering the individual amino acids that make up a protein rather than their average cost, particularly given that these amino acids have different lengths (relatedly is the length of the protein captured in the cost of the amino acid synthesis)?

Formula 1 is the one-time cost incurred regardless of the length of the cell cycle. It is based on the work of Lynch & Marinov (2015). The average cost of AA already takes into account the protein length. Figure 2b shows that the composition in AA of proteins does not affect the rhythmicity. i.e., rhythmic proteins are, in average, not more enriched in costlier individual AAs.

Discussion

- In a number of places, the authors refer to the cost of temporal regulation. This is not quantified by any formulae in the paper. How does this compare to the cost of protein expression? Particularly given the notes here that these calculated costs are based on a steady state.

We haven't quantified costs of regulation as it is difficult to estimate. Here, by cost we not only mean energy costs, but also costs of toxicity as the reviewer also proposes below. We have now clarified this in the main text :

> *This argues that rhythmic regulation has costs, since it is not general. These costs are probably related to the complexity of regulation to maintain precise temporal organisation, and could also be related to toxicity.*
>
> *[...]*
>
> *Finally, rhythmic regulation of highly expressed genes might be driven by other costs than energy, i.e. high expression levels could be detrimental, e.g. because of toxicity.*

In Supplementary Information, we discuss the costs of maintaining a steady-state protein level and explain that rhythmic and highly expressed proteins remain costlier for the cell per time-unit than rhythmic and lower expressed proteins. Therefore, our results remain consistent even if the cost is based on a steady state.

- Do the different phases of genes and proteins make it so that costs are relatively constant across the 24-cycle? Many genes and proteins are active at dusk and dawn – is there an evolutionary hypothesis for these times which would seemingly have concentrated periods of high energy cost?

Although they did not mention it in their paper, Wang et al. (2015) have shown that total energy cost is almost similar in different time-points. Thus, the costs seem to be proportionally spread within the 24 hours.

Questions for Consideration:

Introduction

• The authors argue that the fact that some genes are constant or absence in some tissues

but cycle in others is evidence that rhythmic regulation has costs. They claim this is due to the temporal precision required, but is it actually just costly in the amount of gene/protein produced? In other words, do the tissues with constant levels of the gene/protein produce less over the course of 24 h, and hence the higher cost is actually just more production (rather than the timing specific regulations)?

Indeed, they do. This specific question that takes into account the overall cost over 24 hours has previously been answered by Wang et al. (2015). We have now clarified this in the main text:

> *Wang et al. (2015) have shown that in the liver of mice, abundant proteins that are required at one time are down-regulated at other times, apparently to economize on overall production. Wang et al. also reported that at each time-point, the total metabolic cost was 4 fold higher for the set of cycling genes compared to the non-cycling genes set at both transcriptional and translational levels (Wang et al. 2015) – although the proteomic data used from mouse fibroblasts appear to have been underestimated and have since been corrected (Schwanha ̈usser et al. 2013).*

We thank the reviewer for allowing us to clarify this point.

• Are additional mechanisms required to prevent rhythmicity at the protein level when RNA is rhythmic? How do the costs of preventing rhythmicity in these cases compare to the costs of rhythmicity?

In this case, the translational activity needs to rhythm in anti-phase. Thus, gene expression would need to temporally coordinate the anti-phase activities of the transcription and the translation, in order to finally obtain a constant protein level. To know this, we need to measure the variation of transcriptional and the translational activity, in addition to the mRNA and protein level. To our knowledge, there is no such data available for the same organ in the same species.

Cyclicality of costliest genes

- It seems to me that the discussion of greater costs for higher expression levels of proteins is central to the authors' arguments and should be moved to the main text?

  It seems to us this is already within our main discussion. For instance, it is discussed in the section "Rhythmic proteins: reduction of overall cost".

  > *Indeed, the costs of maintaining a steady state protein level are higher for highly expressed than for lowly expressed genes, even if higher expressed proteins have longer half-lives (see section 4 in Supporting information).*
  >
  > *Our results also suggest that rhythmicity at the protein level only minimizes costs within the constraints of the rhythmic proteins, without making these costs similar or smaller than those of constant proteins.*

- It may be valuable to consider a 3 dimensional plot of expression level vs. length vs. cost to better show that the cost is being driven by expression more than length.

  It is often difficult to interpret 3D plots, and we would prefer to keep it in 2D.

  Genes tend to be rhythmic in the tissues in which they are highly expressed

- How are the expression levels determined? Do you compare total expression, mean expression, or expression at the peak? Have the p-values been appropriately adjusted for multiple comparisons?

  In this case, the gene expression level is the mean expression normalized by Z-score transformation (described in Method formula 7). As we did only one comparison, no adjusting was needed.

  > *For each gene, we estimated the difference δ of expression levels between these two groups of tissues (see Formula 7 in Methods)*

- It would help to at least refer to the methods section here for a definition of tau.

  > *For the mouse circadian dataset (transcriptomic) with 11 tissues, partial correlations show that genes whose rhythmicity is tissue-specific have tissue-specific expression (τ, Formula 8, versus number of rhythmic tissues:*

- Lower cell-to-cell variability of genes with rhythmic transcripts. How is the timepoint at which noise is assessed chosen? There are a number of claims here that are not particularly clear as to why this is generalized?

  This noise data come from an unknown time-point. This is the reason why we discuss those limits:

  > *However, our results are limited by the fact that noise estimation is based on a single*

time-point measurement since no scRNA time-series data are currently available for these species. Since the peak time of rhythmic transcripts is distributed across all times (Supporting Information Fig. S9a), the mean noise estimated at a given time-point includes the noise of the genes that are peaking at that time (lowest noise) and all the others that have a higher noise than those at their own peak time-point (Supporting Information Fig. S9b). Our results suggest that rhythmic genes peaking at the time-point of the scRNA measurement have sufficiently low noise for the mean noise of rhythmic genes to be much lower than that of non-rhythmic genes.

Our results show that noise is globally reduced for genes with rhythmic regulation at the transcriptional level. Since rhythmic genes are not all in the same phase (Fig. S9a in Supporting information), we expect this result obtained for a given time-point (noise estimation based on a single time-point scRNA dataset) to be general to all time-points (section 6.3 in Supporting information). Assuming that genes with low noise have noise-sensitive functions (and thus noise is tightly controlled), these results suggest that rhythmic genes have their noise periodically and drastically reduced through periodic high accumulation of their mRNAs.

- Relatedly, in comparing noise – are differences in expression do to differences in amplitude, phase, or both?

The phase and amplitude are parameters we haven't studied here as we concentrated on the rhythmic versus non-rhythmic nature of the gene expression.

Discussion

- Have you considered genes/proteins with ultradian rhythms? Or is there something

unique to the circadian length cycle that results in evolutionary advantage? Is there a difference in genes/proteins that can be classified as circadian (endogenous) and others? How many of the genes/proteins are downstream of the core circadian clock and does that explain the rhythmicity?

On the basis of our hypothesis, we have only studied rhythms with a 24h period. Some ultradian rhythms are also nycthemeral (12h, 8h, …), but their detection is a matter of specific detection methods. Importantly, we are interested in all genes which rhythm, not only those which are circadian (i.e. that they rhythm by themselves), which are a well studied and quite restricted set. We do not have information at present on which of the rhythmic genes are downstream of the core circadian clock vs. entrained by other mechanisms, although that would be in principle interesting to study in the future.

- Are there additional experiments that could be proposed to test your theory? For example, thinking about the toxicity of proteins that oscillate or that levels are needed for protein-protein interactions? Or ways to manipulate RNA expression to more directly test for noise effects?

This is an interesting question, but it is out of the scope of our study. An experiment that could be done would be to measure the cell-to-cell protein fluorescence (to have the protein variation) at different time-point for different tissues and species. To test the toxicity, one could assess it by measuring the fitness effect of noise based on growth rate in bacteria. However, to really see fluctuations, we might need to be able to follow real-time fluctuations of the fluorescence, compare it between cells, and see the differences in growth rates.

Materials and Methods

- How do you account for genes/proteins that have a period of near 24 h but not exactly 24

h? Do you renormalize to make comparisons?

Here we used GeneCycle method to detect rhythms in gene expression as we have shown in our previous benchmark that it is one of the best methods. It generally accepts an error of 1-2h. Thus we accepted such periods of near 24h which were detected by GeneCycle, to classify genes as rhythmic.

Minor Comments and Questions:

Introduction

• I don't understand what the clause "translation is the major contribution to making expression costs visible to selection" means. Why are some processes "invisible" to selection? What does it mean to be "visible"? Do you mean easily quantifiable?

"Visible to selection" means it changes the phenotype enough to give the individual an advantage or a disadvantage in its environment. In general, for an efficient population size of N_e, it means that the force of selection s is such that $s \cdot N_e >> 1$.

Figure 1b

• I don't understand the connection between the middle and right panels. The middle shows a single protein of each class, and the right shows a box plot – where does the variation in the box plot come from? How is daily contribution to fitness calculated and where does the cost (very clearly illustrated in the middle panel) factor in?

The boxplot shows the expectation from populations of genes behaving like the two examples in the middle. We have now clarified this in the legend

> *b) Rhythmic protein regulation presents a trade-off between the costs generated by integration into the rhythmic system (costs of complexity) and the advantages provided plus the costs saved over 24 hours. The middle exemplifies two extreme behaviors, while the right shows the distribution expected from populations of genes which follow these behaviors.*

Figure 1c

• It would be helpful to overlay a narrow and flat fitness function to show the relationship between the two curves – this makes intuitive sense but showing the match between the peak protein expression and the shape of the fitness function would make this relationship more explicit.

This is difficult to do in 2 dimensions, as fitness would have to be a third dimension.

Genes tend to be rhythmic in the tissues in which they are highly expressed

- Please include which test is being used for rhythmicity with the p-value.

  > *For each gene we separated the tissues into two groups, those for which the gene was rhythmic (Rhythmic detection method, GeneCycle: p-value≤0.01) and those for which it was not (Rhythmic detection method, GeneCycle: p-value>0.5).*

- I understand the motivation for ignoring intermediate p-values, but how did you choose the p-value cutoffs? Is the significance of your results sensitive to the chosen cutoff?

  We chose cutoffs based on the results we obtained in our previous work where we have shown that only genes (for transcriptional rhythmicity) with a strong rhythmic signal, i.e. at least *p*-value≤0.01, should be considered as relevant. Furthermore, genes are often distributed with a

peak near to *p*-value=1. Thus, we have chosen an upper cutoff of *p*-value>0.5 to remove genes that are classified as intermediary by the algorithm, as well as to be able to consider that they have no evidence of rhythmicity with confidence.

Minor Edits and Corrections:

- Cyclicality of costliest genes

Move equation 1 to appear before "other costs such as AA polymerization ..." to be

easier for the reader so that the notation and formula appear close together Figure 1

Thank you, we modified it.

• Consider moving the panel labeling to appear at the upper left rather than lower left. For example, in reading the caption, it was hard to tell whether what is supposed to be panel b was part of panel a.

We modified it.

Discussion
• "but as *be* a by-product of the selection" – remove this extra word typo

We modified it.

Materials and Methods

- Title of this section should be Materials *AND* Methods
- In papio anubi, "time-serie***s***" – add the "s"

We modified it. Thank you.

The author compared the expression levels of rhythmic and non-rhythmic proteins, and concluded that the abundance of rhythmic proteins in arabidopsis, mouse and ostreococcus was higher than that of non-rhythmic proteins, and further calculated the energy consumption of proteins according to the protein abundance, and found that the cost of rhythmic proteins was higher than that of non-rhythmic proteins.

Major comments

1) I am not an expert in proteomics, but according to my knowledge, the proteomics data of mouse liver and cyanobateria used by the authors used SILAC and TMT for protein quantitation respectively, both of which are relatively quantitative methods. Because of the different ionization efficiencies of different peptides, it is not possible to directly compare MS signals of different peptides to determine their abundance in samples, but peaks with the same peptide can be compared in different samples (PMID: 30609196). I checked the data and code using to calculate mouse liver data from Mauvoisin et al., which was used by the author. Although the calculation code was provided, the annotation was not very clear. I think the author used Ratio H/L as the expression of protein abundance. However, Ratio H/L refers to the intensity of each peptide relative to the ratio of the same peptide in the MIX group, which cannot be used for comparison between different proteins. The current relative quantitative method that can be used for comparing proteins can be quantified by label free, processed by MaxQuant, and compared with the IBAQ value therein.
Indeed, the reviewer is correct, and we thank them for pointing this out. After consultation with proteomics specialists (see updated acknowledgements), we have updated all analyses which

compare protein expression with each other in mouse. We obtained similar results, with slightly lower p-values.
We have updated: Fig 2a, Fig 2d, Fig 2e, and Fig S2 (Mouse)

2) The authors calculated the energy expended in protein translation, but not in RNA transcription. The authors assume that the energy cost of RNA transcription is negligible compared to that of protein translation. This is possible in terms of calculating the total energy of transcription and translation, but on the other hand, transcription takes place in the nucleus, and its energy use might be thought of as being in a relatively closed system. Is it possible to calculate the energy of RNA transcription alone? Do the authors think such calculations have biological significance? If agree, I suggest that this work be supplemented.
Our estimation of expression costs is based on the work of Lynch and Marinov (2015) and Wagner (2005). They have studied and reported these points in detail in their work. We have replicated their results and have used them to understand rhythmicity, which is the focus of our manuscript. We improved the calculation by using a more recent dataset and show that expression costs at the protein level are at least 160 times higher than at the RNA level (section 3 in Supporting information).

We now mention this in Methods:

> *In mouse, we have used peptide level data to compare protein abundances in the calculation of expression costs.*

3) In the absence of other data, the authors have to assume that this noise calculated at the transcriptional level is representative of noise at the protein level, or in any case functionally relevant. I don't think this is a reasonable estimate because the error is too large to make the protein noise calculated using this assumption meaningful. Perhaps the authors should have used other methods to estimate the amount of protein and protein noise from single cell data, for example, ribosome profiling. Or the author can discuss the error of the estimation.
Thank you for pointing out this. To our knowledge, the transcriptional noise is one of the main driver of the overall noise, at least in bacteria.

> *Transcription rather than translation seems to be the main source of overall expression noise (section 6.1 in Supporting information)*

Therefore, it should give a relevant estimate of the overall noise. The best way to assess the "functional noise" would have been to catch the cell-to-cell variability at the protein level (by fluorescence for example). Unfortunately, there is no such data for those species and tissues.

We have now clarified this by specifying:

> *Thus, periodic accumulation of mRNAs might be a way to periodically reduce transcriptional noise of noise-sensitive genes*
>
> *Transcription rather than translation seems to be the main source of overall expression noise (section 6.1 in Supporting information). However, we can only conclude regarding the transcriptional noise.*

Minor comments

1) To test the hypothesis that genes tend to be rhythmic in the tissues in which they are highly expressed, the authors used a mouse circadian dataset with 11 tissues (transcriptomics from Zhang et al. (2014), Supplementary Table S2). As far as I know, that dataset includes a total of 12 mouse tissues. What about the other tissues? If a selection was made, perhaps the author should state the criteria in the data section.
Indeed, we chose to ignore the hypothalamus as there are almost no rhythmic genes in this tissue, which contains the Supra-Chiasmatic Nucleus in which the rhythmic genes are the core clock genes

that synchronize the other tissues. Therefore, their rhythmicity cannot be due to a regulation of cost. Moreover, the constant expression of other genes is assumed to be essential for brain function.

We now mention this in Methods:

> *We have volontarily excluded the hypothalamus since it contains the Supra-Chiasmatic Nucleus known to have core clock genes that synchronise the body. Therefore, their rhythmicity is probably dominated by other causes than a regulation of cost.*

2) In this paper, gene expression noise is a very important concept. Although the author directly uses the calculation method by Barroso et al. (2018) when quantifying noise expression, I think it is still necessary to explain the quantification process of noise and the biological significance of noise in more detail in the method section.
In the Supporting Information document, we describe the method we used to estimate the gene expression noise in section 6.4 “Noise estimation”. We compared 8 methods and provided the results of the slope calculation of the linear model which best fit the correlation between the noise and mean expression, as well as the R2 and the Kendall correlation applied to normally distributed noise estimations.

> *The linear model that explained the variance the least well (small R2) was considered to be approximately the best expected value. Indeed, we expect a good noise estimation model to be independent of the mean gene expression (linear regression slop and Kendall’s τ near to 0) and with a large range of noise values for every mean expression (small R2).*

We have now added the following precisions to the Methods section in “Gene expression noise quantification”:

> *We tested different degrees of the polynomial regression to estimate log(σ2) in the calculation of F_* and measured the correlation based on Kendall’s rank and linear regression slope (see Supporting information section 6.4). The F^* polynomial degree of Barroso et al. (2018) was the best method because it removed the correlation between mean expression and F^* (smallest linear regression slope and Kendall’s τ) and keep a large range of noise values for every mean expression (smallest R2) (Supporting information section 6.4). Finally, since the peak times of rhythmic genes is largely distributed (Fig. S9a), we expect the mean noise of a given time-point to be general to all time-points (Fig. S9b), therefore giving a good indicator to estimate its biologically relevant.*
