# Supplementary material for: Rhythmicity is linked to expression cost at the protein level but to expression precision at the mRNA level
Source: PLoS Comput Biol. 2022 Sep 12;18(9):e1010399. doi: 10.1371/journal.pcbi.1010399 (PMC9518874; doi:10.1371/journal.pcbi.1010399)
Supplement: Attachment — Submitted filename: LaloumPlosCompBioReviewRevision.pdf [file pcbi.1010399.s021.pdf]

Review of “Rhythmicity is linked to expression cost at the protein level but to expression precision at the mRNA level”

Response to Review:

We thank the authors for their response to the reviewer comments, which were well addressed in their responses but only few were incorporated into the text. Specifically this should include:

- The authors should explicitly cite that the formula given as been previously developed by Lynch & Marinov 2015, to clarify that validation of the formula is not necessary for its application here.
- Comments on experiments to test their theory. A good theory should be testable, and the authors should propose experiments to test this theory, presenting a valuable way to progress science forward by inspiring new experiments.
- I think it is also worth noting that some of the rhythmicity may result from being downstream of the circadian clock (as alleged in the response to Reviewer 2 about not including hypothalamus).
- The point shown by Wang et al. (2015) that energy costs are evenly spread across the cycle would be interesting to add to the discussion as it relates to the balance of energy costs throughout the day. This would fit neatly with the discussion of the point that cyclic genes have four times the energy cost in the added text.

The authors failed to address the question of whether the significance of their results is dependent on their cutoff for the classification of genes as arhythmic.

- When “intermediate” genes are included are the results still significant? Obviously, including genes that are barely above threshold for rhythmicity will decrease the significance of these results but if the authors had chosen $p>.1$ or $p>.25$ would their findings still hold? Including a supplemental figure showing the distribution of rhythmic values may better motivate this choice of cutoff.

Additional Minor Comments:

Figure 1c

- It is unclear what the black triangles point to in this schematic. As noted in the initial review, relating the ranges drawn on the protein expression curve to the fitness function would be beneficial. This could still be done in 2D by adding a double y axis to the plot.
