# Supplementary material for: Rhythmicity is linked to expression cost at the protein level but to expression precision at the mRNA level
Source: PLoS Comput Biol. 2022 Sep 12;18(9):e1010399. doi: 10.1371/journal.pcbi.1010399 (PMC9518874; doi:10.1371/journal.pcbi.1010399)
Supplement: Attachment — Submitted filename: Response_reviewers.pdf [file pcbi.1010399.s023.pdf]

Reviewer #1:

Response to Review:

We thank the authors for their response to the reviewer comments, which were well addressed in their responses but only few were incorporated into the text. Specifically this should include:

- The authors should explicitly cite that the formula given as been previously developed by Lynch & Marinov 2015, to clarify that validation of the formula is not necessary for its application here.

We have now clarified this in the main text:

> Thus, based on the calculation developed by Lynch & Marinov (2015), we estimated the expression cost (C_p) of each gene by the formula (1) which takes into account the averaged amino-acid (AA) synthesis cost ($\bar{c}_{AA}$) of the protein (Supp Table S1), protein length (L_p), and protein abundance (N_p)

- Comments on experiments to test their theory. A good theory should be testable, and the authors should propose experiments to test this theory, presenting a valuable way to progress science forward by inspiring new experiments.

We have now included this in the Discussion:

> We are aware that this theory is difficult to test experimentally. An interesting direction would be the measurement the cell-to-cell protein fluorescence at different time-point for different tissues and species, providing real-time information on the noise fluctuations at various levels. Moreover, the fitness effect of noise could be estimated from growth rates in unicellular organisms measured at different time-point, under conditions which favor rhythmicity or which don't.

- I think it is also worth noting that some of the rhythmicity may result from being downstream of the circadian clock (as alleged in the response to Reviewer 2 about not including hypothalamus).

We have now clarified this in the main text:

> We call ”rhythmic genes” all genes displaying a 24-hours periodic variation of their mRNA or protein level, or of both, constituting the nycthemeral

transcriptome or proteome. [...] Hence we use the term ”nycthemeral” to avoid confusion with the specific features of ”circadian” rhythms, although these are included in the nycthemeral rhythms, which includes downstream regulated genes.

- The point shown by Wang et al. (2015) that energy costs are evenly spread across the cycle would be interesting to add to the discussion as it relates to the balance of energy costs throughout the day. This would fit neatly with the discussion of the point that cyclic genes have four times the energy cost in the added text.

We noticed that data used by Wang et al. 2015 for the calculation of costs appears to be biased, partly because translation rates come from fibroblasts cells (Schwanhausser et al. 2013), and partly because there were errors in the estimation of protein levels resulting in a systematic underestimation of protein levels and derived translation rate constants (Cf Corrigendum (Schwanhausser et al. 2013)). This is the reason why we deliberately chosen to not include their results as part of our interpretation, but only to use our results to support theirs.

The authors failed to address the question of whether the significance of their results is dependent on their cutoff for the classification of genes as arhythmic.

• When “intermediate” genes are included are the results still significant? Obviously, including genes that are barely above threshold for rhythmicity will decrease the significance of these results but if the authors had chosen p>.1 or p>.25 would their findings still hold? Including a supplemental figure showing the distribution of rhythmic values may better motivate this choice of cutoff.

Yes, we performed the analysis with these cutoffs and the results are the same. Supplementary Figures S10-S12 already provide *p*-value distributions.

Additional Minor Comments:

Figure 1c

• It is unclear what the black triangles point to in this schematic. As noted in the initial

review, relating the ranges drawn on the protein expression curve to the fitness function would be beneficial. This could still be done in 2D by adding a double y axis to the plot.

We have now included two small graphs illustrating flat and narrow fitness functions (Fig 1C)

Reviewer #2:

I am glad that the authors have given detailed replies to my previous comments.

In my last review, I mentioned that the abundance of different proteins obtained by previous quantitative methods cannot be compared with each other. The authors reanalyzed it in this revision. It's nice to see that the new results are slightly different from the previous results that there is no change in the conclusions. However, the authors' reply is not very clear about how to improve the method. I think the authors have moved from analyzing the abundance at the protein level to the peptide level. Is my understanding correct?

Yes we have now used peptide counts.

As for the second question I mentioned earlier, "whether it is necessary to calculate mRNA-related expression energy", the authors calculated that the energy required to produce protein was more than 160 times that of mRNA, and the energy cost of protein was much higher than that of mRNA. Therefore, it makes sense to consider just the protein without needing to be very elaborate. I think the explanation given by the author is reasonable.

As for another comment, that transcriptional noise is not representative of translation noise, I would like to thank the authors for providing relevant article on the fact that transcriptional noise is a major source of noise in bacteria. This substitution makes sense in bacteria and yeast. Unfortunately, there is no data on multicellular species, which would make this paper more rigorous.

The author has answered the reasons why the hypothalamus was not included in the analysis. I think the reasons given are reasonable.

In addition, I have a new suggestion for the author to consider.

Major comments

1)As briefly mentioned in the manuscript, other factors such as protein-protein misinteractions, undesirable enzyme activities or antagonistic pleiotropy may also explain the rhythmicity of gene transcription. The authors should provide strong evidences to exclude the possibility that those factors can lead to noise reduction in rhythmic mRNAs.

From an evolutionary perspective, we tried to understand why did we find low transcriptional noise? And one hypothesis, as the reviewer suggested, is that it was selected because it could reduce protein-protein misinteractions at some time-points for instance. Which is expected to be a sufficiently important advantage to be selected. Reducing noise means reducing variability in a population of cells. We don't find reason to expect that undesirable enzymatic activity would reduce this variability.

To clarify, we have now added this in the main text:

> Finally, **selection for** rhythmic regulation of highly expressed genes might be due to an efficient reduction of periodic protein-protein misinteractions.

And no more other comments.

Have the authors made all data and (if applicable) computational code underlying the findings in their manuscript fully available?

The PLOS Data policy requires authors to make all data and code underlying the findings described in their manuscript fully available without restriction, with rare exception (please refer to the Data Availability Statement in the manuscript PDF file). The data and code should be provided as part of the manuscript or its supporting information, or deposited to a public repository. For example, in addition to summary statistics, the data points behind means, medians and variance measures should be available. If there are restrictions on publicly sharing data or code —e.g. participant privacy or use of data from a third party—those must be specified.

Reviewer #1: None

Reviewer #2: None

The code and the data used in this paper have now been updated and added into the GitHub repository laloumdav/cost_noise_conservation_rhythmicity.

Figure Files:

While revising your submission, please upload your figure files to the Preflight Analysis and Conversion Engine (PACE) digital diagnostic tool, https://pacev2.apexcovantage.com. PACE helps ensure that figures meet PLOS requirements. To use PACE, you must first register as a user. Then, login and navigate to the UPLOAD tab, where you will find detailed instructions on how to use the tool. If you encounter any issues or have any questions when using PACE, please email us at figures@plos.org.

Data Requirements:

Please note that, as a condition of publication, PLOS' data policy requires that you make available all data used to draw the conclusions outlined in your manuscript. Data must be deposited in an appropriate repository, included within the body of the manuscript, or uploaded as supporting information. This includes all numerical values that were used to generate graphs, histograms etc.. For an example in PLOS Biology see

here:http://www.plosbiology.org/article/info%3Adoi%2F10.1371%2Fjournal.pbio.1001908#s5.

The code and the data used in this paper have now been updated and added into the GitHub repository laloumdav/cost_noise_conservation_rhythmicity.

Reproducibility:

To enhance the reproducibility of your results, we recommend that you deposit your laboratory protocols in protocols.io, where a protocol can be assigned its own identifier (DOI) such that it can be cited independently in the future. Additionally, PLOS ONE offers an option to publish peer-reviewed clinical study protocols. Read more information on sharing protocols at https://plos.org/protocols?utm_medium=editorial-email&utm_source=authorletters&utm_campaign=protocols

References:

Review your reference list to ensure that it is complete and correct. If you have cited papers that have been retracted, please include the rationale for doing so in the manuscript text, or remove these references and replace them with relevant current references. Any changes to the reference list should be mentioned in the rebuttal letter that accompanies your revised manuscript.

If you need to cite a retracted article, indicate the article's retracted status in the References list and also include a citation and full reference for the retraction notice.

In compliance with data protection regulations, you may request that we remove your personal registration details at any time. (Remove my information/details). Please contact the publication office if you have any questions.
