# Supplementary material for: Rhythmicity is linked to expression cost at the protein level but to expression precision at the mRNA level
Source: PLoS Comput Biol. 2022 Sep 12;18(9):e1010399. doi: 10.1371/journal.pcbi.1010399 (PMC9518874; doi:10.1371/journal.pcbi.1010399)
Supplement: Attachment — Submitted filename: 116569_1_rebuttal_2174993_qzdq7q-1.docx [file pcbi.1010399.s017.docx]

**Manuscript number:** RC-2021-00776

**Corresponding author(s):** David Laloum, Marc Robinson-Rechavi

## General Statements

We thank reviewers for helping us clarify our manuscript. Some key information was only in the Supporting Information document, and was not obvious to find. We have now introduced some of this information into the main text, and otherwise clarified to which specific sub-paragraph of the Supporting Information document we refer every time we mention it. Another aspect which we have clarified is the relevance of controls previously published in our paper PLOS Comp Biol 16: 1-23. These controls address many of the remarks raised by the reviewers, regarding for instance rhythm detection methods, detection threshold, the effect of normalization of time-series data in rhythm detection, the consideration of biological replicates in time-series data, or the relationship between rhythms and highly expressed genes. We have now introduced some of these results within the main text to clarify these points, or have specified to which specific result of our previous paper we refer.

We also provide a second version of the main text, supporting information and supplementary figures in which we have highlighted new contents or modifications.

## Point-by-point description of the revisions

## *Purple Italics: old text*

*Black Italics: new text*

# **REVIEWER #1**

## **Major comments:**

They assumed the optimal constant level would be the maximum over the rhythm period when rhythmic regulation is absent. They also assumed the trade-off between the benefits of not producing proteins when they are not needed (costs saved) and the costs involved in making it rhythmic (costs of complexity), which they argued lead to the expectation that costlier genes be more frequently rhythmic. However, there was no explicit definition for the trade-off, so it is unclear how it leads to the expectation. [...]

Second, the "costs of complexity" were not defined

We have now clarified these points:

*Thus, a first evolutionary advantage given by rhythmic biological processes would be an optimization of the overall cost (over a 24-hour period), compared to a constant expression at a high level of proteins, when this high level is necessary for fitness at least at some point of time.*

- *Thus, a first evolutionary advantage given by rhythmic biological processes would be an optimization of the overall cost (over a 24-hour period), compared to the costs generated over the same period by optimizing a constant level of proteins.*

*The reasonable assumption that the optimal constant level would be the maximum over the rhythm period strengthens the case for selection on expression cost.*

- *Our results suggest that rhythmicity of protein expression has been favored by selection for cost control of* gene expression, while keeping optimal expression levels. In the case of rhythmic genes, what *would that optimal constant level be? We can propose two hypotheses. The first is that it would be the mean expression over the period, since this maintains the same overall amount of protein. The second is that it would be the maximum over the rhythm period, since that is the level needed at least at some point. The second hypothesis explains better the existence of this maximum level during the cycle. Of note, it also strengthens the case for selection on expression cost. Thus, for the case of rhythmic genes, the optimal constant level should at least correspond to the mean expression level (Fig 1d). We provide results obtained using both the maximum and the mean of expression in Fig. 2a.*

We have modified Fig. 1d accordingly, and specified in Supp Fig. S2 that the delta value was calculated from mean expression levels*.*

*We assume that the maximal expression level gives an estimation of the level that would be constantly maintained in the absence of rhythmic regulation*

- *We assume that, in the absence of rhythmic regulation, the constant optimal level is included between the mean and the maximum expression level observed in rhythmic expression.*

*Here, we studied the evolutionary costs and benefits that shape the rhythmic nature of gene expression at the RNA and protein levels. For this, we analysed characteristics we presume to be part of the trade-off.*

- *Here, we studied the evolutionary costs and benefits that shape the rhythmic nature of gene expression at the RNA and protein levels. For this, we analysed characteristics we presume to be part of the trade-off determining the rhythmic nature of gene expression between its advantages (cost economy over 24h, non-ribosomal occupancy) and disadvantages (costs of complexity related to precise temporal regulation).*

*The evolutionary origin of maintaining large cyclic biological systems, in term of adaptability, can be seen as a trade-off between disadvantages such as cost or noise induced by the added complexity, and advantages such as economy over a daily time-scale, temporal organization, or adaptability.*

- *Most rhythmic genes are tissue-specific (Zhang et al. 2014, Boyle et al. 2017), which means that their rhythmic regulation is not a general property of the gene and is therefore expected to be advantageous only in those tissues in which they are found rhythmic. This argues that rhythmic regulation has costs, since it is not general. These costs are probably related to the complexity of regulation to maintain precise temporal organisation. Thus, cyclic biological systems are expected to have adaptive origins.*

It would be more convincing to define a fitness function or cost function to demonstrate their argument that costlier genes have fitness advantages if they are rhythmic.

Considering rhythmicity as an economy strategy is quite intuitive and our results confirm what is currently accepted (Wang et al. 2015). We show and discuss to which extent this is true by comparing expression costs at different expression levels. Defining more precisely a fitness function in our case would require an experiment where we could compare fitness between two populations (e.g. prokaryote growth rates): WT versus a strain whose promoter of the costliest genes would be controlled by non-cyclic transcriptional factors. We do not feel that this is a reasonable extension of this work, but a whole new research program.

First, when proteins are not needed, it can be either the case of not producing extra proteins (cost saved) or the case of degrading excessive proteins (cost incurred). […]

The cost function presented in this paper may be oversimplified. It only takes into account the costs to produce protein. The authors argued that a more complex cost calculation would not change the observation, but without proving it. However, protein degradation, including ubiquitination and proteolysis, requires energy; for a rhythmic gene, it is also necessary to consider the cost of maintaining the rhythmicity, including the temporally precise regulation of protein expression when the proteins are needed and of protein destruction when they are not.

We have now clarified this in Section 4.1 of the Supporting Information document:

Protein decay can be due to spontaneous decay of unstable molecules (no cost), cellular dilution (no cost), or active protein degradation, which has a cost which has been shown to be negligible. Costs of protein decay are negligible enough to not be opposed by selection. Indeed, Lynch and Marinov (2015) and Wagner (2005) have shown that “*degradation in a lysosome may cost essentially nothing, and amino-acid export back to the cytoplasm consumes ∼1 ATP for every 3 to 4 amino acids*”. Compared with the unique cost of producing one single nucleotide which consume 49~P, protein decay costs becomes negligible comparatively to transcriptional costs, which are themselves negligible comparatively to translational costs. All the more, given that amino acids from degradation are reused and do not need to be produced by the cell, which therefore economizes around 30 ~P per amino-acid (~P: high-energy phosphate bonds).

In Section 3 of the Supporting Information document, we also show why rhythmic and highly expressed proteins are costlier for the cell per time-unit than rhythmic and lower expressed proteins, even considering decay costs or proteins half-lives.

Thus, the order of costs between genes is not expected to be affected by a more complex calculation accounting for protein decay and protein half-lives.

We think these points should be in Supporting Information document since they are not novel. Lynch and Marinov as well as Wagner have studied and reported these points in detail in their work. We have replicated their results and have used them to understand rhythmicity, which is the focus of our manuscript.

The authors claimed that cycling genes are enriched in highly expressed genes, by showing rhythmic proteins are costlier than non-rhythmic proteins (based on the expression cost function) in several species. However, only the first 15% of proteins based on p-values ranking from their rhythm detection algorithms were classified rhythmic. One potential artifact of this classification is that the identified rhythmic genes are biasedly highly expressed genes because the lower-amplitude genes are harder to detect and excluded by the algorithm. If changing the threshold for rhythmicity to include more rhythmic genes with intermediate p-values (p-value<=0.05), will this change the results?

Since the results of this paper would be sensitive to the accuracy of identifying rhythmicity at both mRNA and protein levels, it is crucial to validate the rhythm detection algorithm by cross-checking algorithm-generated results with those known rhythmic genes. Can the authors estimate the false positive and false negative rates in each group of the rhythmic and non-rhythmic proteins or mRNAs identified by their algorithm?

Our 2020 paper (*https://journals.plos.org/ploscompbiol/article?id=10.1371/journal.pcbi.1007666*) addresses these issues, but we did not make this sufficiently clear here. We have now added some details of our previous results in the main text to clarify, as this a logical limitation remark. We mostly use GeneCycle based on the results of the benchmarking in that paper; it notably produces a uniform distribution under the null hypothesis and a skew towards low p-values for all empirical data.

*Furthermore, cycling genes have been shown to be over-represented among highly expressed genes (Laloum & Robinson-Rechavi 2020, Wang et al. 2015).*

- *Furthermore, we have shown in our previous work that rhythmic genes are largely enriched in highly expressed genes, and that the differences in rhythm detection obtained between highly and lowly expressed genes either reflect true biology or a lower signal to noise ratio in lowly expressed genes (Laloum & Robinson-Rechavi 2020).*

Higher gene expression usually leads to lower genetic noise. The authors thus applied a definition of the stochastic gene expression (SGE) that controls the biases associated with the correlation between the expression mean and variance to evaluate expression noise. They found lower noise with rhythmic transcripts. However, they did not explain, mechanistically, why rhythmic RNA has lower noise and what is the biological meaning behind this finding. It is also unclear whether they considered the phase difference between signal and noise that usually exists in an oscillatory system.

Please see answer to second reviewer

## **Minor comments:**

It would be helpful if the authors could interpret their observations including where the results may not be as significant. A few examples are listed below.

1) In tissue-specific studies, they used the transcriptomics datasets from 11 mouse tissues to compare the difference in expression levels (based on z-score) of each gene between tissue groups of rhythmic and non-rhythmic expression and found higher gene expression in rhythmic tissues. However, proteins showed a bimodal distribution, and it would be helpful to add interpretation or discussion regarding this bimodal distribution.

Note that for proteins, the delta was calculated based on only 3 or 4 tissues, which limits a lot our detection power. We now proposed the hypothesis:

- *We also provide results obtained from other datasets in supplementary Table S3, although they must be taken with caution since only 2 to 4 tissues were available, and sometimes data were coming from different experiments. Of note, for proteomic data, the distributions of* ẟ *are bimodal (Fig. S3), separating rhythmic proteins into two groups, with low or high protein levels in the tissues in which they are rhythmic. A hypothesis is that for some tissue-specific proteins the rhythmic regulation is not tissue-specific, making them rhythmic also in tissues where they are lowly expressed. But the very small sample size does not allow us to test it, and we caution against any over-interpretation of this pattern before it can be confirmed.*

2) They also calculate partial correlation for rhythmicity with expression level over tissues for all tissue-specific genes (tau>0.5) and found Spearman's correlation coefficient is skewed towards negative (suggesting a correlation), but Pearson's correlation showed a positive peak. It indicates that a subset of genes is less rhythmic in the tissues where they are most expressed. Is this positive peak significant or expected? What are these genes? Any evolutionary benefits? Can the authors discuss the functional difference between these genes and other genes that follow the predictions?

*While Spearman’s correlation is clearly skewed towards negative correlations, i.e. lower p-values thus stronger signal of rhythmicity in the tissue where genes are more expressed, Pearson’s correlation also has a smaller peak of positive correlations (Fig. S4), suggesting a subset of genes which are less rhythmic in the tissues where they are most expressed.*

- *While Spearman’s correlation is clearly skewed towards negative correlations, i.e. lower p-values thus stronger signal of rhythmicity in the tissue where genes are more expressed, Pearson’s correlation also has a smaller peak of positive correlations (Fig. S4a), suggesting a subset of genes which are less rhythmic in the tissues where they are most expressed. We show that tissue-specific genes which are mostly rhythmic in tissues where they are highly expressed are under stronger selective constraint than those which are rhythmic in tissues where they are lowly expressed (Fig. S4b). Thus, rhythmic expression of this second set of genes might be under weaker constraints.*

*We added Fig. S4b in Supplementary figures.*

3) In SGE analysis, the scRNA data of Arabidopsis was from roots, while the data for detecting the rhythmicity was from leaves. Without knowing whether the gene expression patterns in these two different parts are comparable, it is hard to judge the results. The authors may want to provide some discussion.

Indeed, this limits the interpretation for Arabidopsis, as noted in the results and in the discussion. We still prefer to report this pattern than to remove it. But, we have now moved the results obtained for Arabidopsis into Supplementary Table S5.

- *In Arabidopsis, the single-cell data used are from the root, while transcriptomic time-series data used to detect rhythmicity are from the leaves, which limits the interpretation. Despite this limitation, we found no evidence of lower noise for genes that are rhythmic at the protein level (Table 1b and 1e, and Supplementary Table S5), and trends towards lower noise in almost all cases for genes with rhythmic mRNAs (Table 1a, 1c, and 1d).*
- *Our results in mouse are consistent with all of these considerations (Table 1 and Supplementary Table S5), although it was not fully the case for Arabidopsis (Supplementary Table S5). However, this last point might be explained by the tissue-specificity of rhythmic gene expression. Indeed, for Arabidopsis, the time-series dataset come from leaves whereas single-cell RNA data come from roots.*

For Mouse tissues, while most show lower noise for rhythmic genes, they saw the opposite in Muscle. Is this significant? Any discussion?

For mouse muscle, we had not mentioned it since it was the only tissue showing such a trend. We now added comment regarding this in the main text:

- *In mouse, tissue muscle gave opposite result, possibly because skeletal muscle is one of the most un-rhythmic tissues in the body.*

In various places of the text, the authors only pointed the readers to "Supporting information" without explicitly referring to a specific supplemental figure by its number. It would be helpful to cite a table or figure explicitly.

We agree, and have corrected this. See first General Statements.

Figure 2 does not have legends in the graphs.

This is now corrected, thank you for your attention

# **REVIEWER #2**

## **Major comments:**

- Our major concern regards the identification of rhythmic genes.

Despite we are not experts in the specific method used (details are not provided in the manuscript), a method looking for a statisical significant periodicity in a noisy signal will provide a high p-value for a signal sufficiently above the noise level. Gene expression data are noisy because of stochastic gene expression and technical noise (e.g., the sampling noise due to RNA capture in RNAseq data). This noise scales with the average level of expression. Lowly expressed genes generally display larger relative fluctuations (e.g., sampling noise is essentially Poisson-like). As a result, the method will identify with a higher probability genes that are highly expressed as rhythmic genes since the signal to noise ratio is generally higher.

This could significantly bias the subsequent analysis, since most of the claims are related to a link between expression levels and rhythmicity.

[There is not even an obvious separtation of timescales that can be invoked between a possible 24-hour periodic signal and the fluctuations. For example, the timescale of protein fluctuations can be largely set by dilution and thus have a timescale comparable to the cell cycle.]

The authors should discuss this issue, which is overlooled in the current manuscript.

How much this potential bias affects the selection of rhythmic genes can probably be assessed using synthetic data.

- It would be useful to clarify in the main text what are the units of measurement of gene expression at the mRNA and at the protein level. If we understood correctly, the authors used FPKM and protein counts respectively. The dynamics in time could in principle be different if an absolute or a normalized level of expression is considered. For example, the cell cycle can be correlated with the circadian clock (as reported for example in cyanobacteria). Since the absolute amount of total proteins has to approximately double during a cell cycle (for cell size homeostasis), this can create a periodic signal in protein counts with a 24-hour period.

The same reasoning does not hold true if the measurement is normalized, as in the FPKM case.

The authors should discuss this issue or simply show that the results for proteins are robust if the protein count is normalized (for example with respect to the total protein amount).

We haven’t focused the present manuscript on these issues since we recently published another paper which addresses these points: [*https://journals.plos.org/ploscompbiol/article?id=10.1371/journal.pcbi.1007666*](https://journals.plos.org/ploscompbiol/article?id=10.1371/journal.pcbi.1007666)

We have now added some details of our previous results in the main text to make the work more relevant.

- The expression cost defined in the manuscript seems dominated by the expression level.

It would be useful to report the scatter plot and the correlation level of cost versus average expression. A high correlation between these two quantities can largely recapitulate the results in Figure 2 (even though the results presented are still interesting per se). In other words, the relation between cost and rhytmicity sounds like a simple rephrasing of the relation between average expression level and rhythmicity (previously reported as correctly referenced in the manuscript).

We now provide these results in Fig. S2 (Supplementary figures) and show a negative and significant correlation between the order of the rhythmicity signal and the total expression cost (calculated from the mean expression level). Since our previous benchmark show that the order of genes from most to less rhythmic genes is not very reliable for known methods, including the one used here, we prefer to present this result in the Supplementary figures document.

- The empirical observation of a relation between noise and rhythmicity in mRNA expression is interesting, but we cannot fully understand its link with the theoretical arguments proposed.

The Authors suggest that perodicity in mRNA expression could decrease protein noise at the peak of mRNA expression (Fig.S1). But this is not what they can measure in the single-cell data analyzed, where cell-to-cell variability is reported at a single timepoint for a cell population. If the oscillations are not syncronized in the cell population, an oscillating transcript would simply display a high cell-to-cell variability dominated by the amplitude of oscillations. Even if the oscillations are syncronized, there is no information in the dataset about the mRNA dynamics. Thus, mRNA cell-to-cell variability could have been measured at any point of its (putative) cyclic dynamics.

Thus, we propose to make more clear the connections between the theoretical arguments and the empirical observation about noise in gene expression.

## Thank you for pointing out this issue. We have clarified the following in the main text:

*These considerations lead to predictions which we test here: i) a decreased stochasticity strategy for genes with rhythmically accumulated mRNAs ....*

- *These considerations lead to predictions which we test here: i) a strategy to periodically decrease stochasticity for genes with rhythmically accumulated mRNAs ....*

*Assuming that genes with low noise have noise-sensitive functions (and thus noise is tightly controlled), these results support the hypothesis that noise is globally reduced thanks to rhythmic regulation at the transcriptional level.*

- *Our results show that noise is globally reduced for genes with rhythmic regulation at the transcriptional level. Since rhythmic genes are not all in the same phase (Fig. S9a in Supporting information), we expect this result obtained for a given time-point (noise estimation based on a single time-point scRNA dataset) to be general to all time-points (section 6.3 in Supporting information). Assuming that genes with low noise have noise-sensitive functions (and thus noise is tightly controlled), these results suggest that rhythmic genes have their noise periodically and drastically reduced through periodic high accumulation of their mRNAs.*
- *Thus, since we find lower noise among rhythmic transcripts, rhythmic expression of RNAs might be a way to periodically reduce expression noise of highly expressed genes (Figure 2 and Fig. S1-S2), which are under stronger selection. Indeed, we found that genes with rhythmic transcripts are under stronger selection, even controlling for expression level effect. As proposed by Horvath et al. (2019) and supported by results in mouse by Barroso et al. (2018) genes under strong selection could also be less tolerant to high noise of expression. Thus, periodic accumulation of mRNAs might be a way to periodically reduce expression noise of noise-sensitive genes (Fig 1c), i.e. genes under stronger selection. However, our results are limited by the fact that noise estimation is based on a single time-point measurement since no scRNA time-series data are currently available for these species. Since the peak time of rhythmic transcripts is distributed across all times (Supporting Information Fig. S9a), the mean noise estimated at a given time-point includes the noise of the genes that are peaking at that time (lowest noise) and all the others that have a higher noise than those at their own peak time-point (Supporting Information Fig. S9b). Our results suggest that rhythmic genes peaking at the time-point of the scRNA measurement have sufficiently low noise for the mean noise of rhythmic genes to be much lower than that of non-rhythmic genes.*

- As a simple additional test of robustness of the rhythmic gene selection, biological replicates can be used, although this would not resolve the possible bias discussed above. As explained by the Authors, some of the datasets analyzed have biological replicates. It would be interesting to know the robustness of the detection method across replicates. How much is the set of genes identified as rhythmic conserved if estimated on different replicates? Spearman correlation or simply the overlap between the sets (maybe assessed with a hypergeometric test) can be used.

These points have been already addressed in our 2020 paper [*https://journals.plos.org/ploscompbiol/article?id=10.1371/journal.pcbi.1007666*](https://journals.plos.org/ploscompbiol/article?id=10.1371/journal.pcbi.1007666) (paragraph “The importance of having an informative dataset”) as well as in recent guidelines (Hughes et al. 2017). We specified in Methods that we considered replicates as new cycles as recommended.

## **Minor comments:**

- The claim that "transcriptional noise is known to be the main driver of overall expression noise", which is present in the discussion is questionable.

For example, the quantitative large-scale dataset referenced by the Authors for E.coli (Taniguchi et al) shows instead that the dominant source of noise is extrinsic for many of the genes tested.

We have clarified in the main text that by “main driver of the overall noise” we refer to the relative contribution of transcriptional versus translational noise into the overall noise.

## We have also added the section 6.1 into Supporting Information document:

- *Relatively to translational noise, transcriptional noise is the main driver of the overall noise (Raj and van Oudenaarden 2008) and should give a good estimation of the output noise. Indeed, based on estimations of coefficient of variations (CV, cell-to-cell variations of protein level) for diverse transcription and translation rates in E. coli and S. cerevisiae, Hausser et al. (2019) have shown that for a fixed transcriptional rate, CV is almost constant for diverse translational rates. Thus, changes in protein level have little to no impact on gene expression noise. The availability of mRNA molecules seems to drive the final noise. I.e., comparatively to the noise caused by the translational activity, the availability of low number molecules such as transcriptional factors (subject to the stochasticity of diffusion and binding in the cell environment) is the main factor of the output cell-to-cell variation in protein abundances.*

And have modified the main text:

*Indeed, transcriptional noise, which we measure here, is known to be the main driver of overall expression noise (Raj & van Oudenaarden 2008).*

- *Relatively to translational noise, transcriptional noise is the main source of the overall noise (Raj & van Oudenaarden 2008) (section 6.1 in Supporting information)*

In addition, highly expressed proteins are all precisely expressed and they display little variation in noise (also shown by Hausser et al. (2019) who reused Taniguchi et al. (2010) data). The noise of these highly expressed proteins is also just above a limit which is the noise floor. This "noise floor" is dominated by extrinsic noise as suggested by Hausser et al. and Taniguchi et al.: “The extrinsic noise in the last three terms in [Eq. 4](https://www.ncbi.nlm.nih.gov/pmc/articles/PMC2922915/#FD4) (of the noise floor) might originate from fluctuations in cellular components such as metabolites, ribosomes, and polymerases and dominates the noise of high copy proteins” (Taniguchi et al.). Thus, highly expressed proteins are precisely expressed and their residual noise is similar to the noise floor, which is due to the extrinsic noise (imperfect synchrony of cell states inherent or due to the environment).

- We suggest to avoid explicit statements about a causal link between expression level and rhythmicity, as in the caption title of Figure 2. A detected correlation is not a proof of a causal relation.

We have corrected the sentence as follows:

*Rhythmic proteins are costly proteins due to their high level of expression.*

- *High level of expression is the main factor explaining the higher cost observed in rhythmic proteins.*

- Supplementary Figures attached at the end of the main text and Supplementary Figures in the Supporting Information file have the same numbering...so there are two different versions of Fig.S1 S2 etc.

This complicates the work of the reader.

We have modified the numbering of figures to make them easier to follow.

-The legend of Fig 2 is missing (the legend is instead reported in Fig.S1).

This is now corrected, thank you for your attention

***Other modifications:***

*We also show how cost can explain the tissue-specificity of rhythmic gene expression. Indeed, the nycthemeral transcriptome has long been known to be tissue-specific (Zhang et al. 2014, Boyle et al. 2017, Korenˇciˇc et al. 2014), i.e. a given gene can be rhythmic in some tissues, and constantly or not expressed in others.*

- *Furthermore, the nycthemeral transcriptome has long been known to be tissue-specific (Zhang et al. 2014, Boyle et al. 2017, Korenˇciˇc et al. 2014), i.e. a given gene can be rhythmic in some tissues, and constantly or not expressed in others. Here, we provide a first explanation for the tissue-specificity of rhythms in gene expression by showing that genes are more likely to be rhythmic in tissues where they are specifically highly expressed.*
