# Supplementary material for: Rhythmicity is linked to expression cost at the protein level but to expression precision at the mRNA level
Source: PLoS Comput Biol. 2022 Sep 12;18(9):e1010399. doi: 10.1371/journal.pcbi.1010399 (PMC9518874; doi:10.1371/journal.pcbi.1010399)
Supplement: Attachment — Submitted filename: comments.docx [file pcbi.1010399.s019.docx]

The author compared the expression levels of rhythmic and non-rhythmic proteins, and concluded that the abundance of rhythmic proteins in arabidopsis, mouse and ostreococcus was higher than that of non-rhythmic proteins, and further calculated the energy consumption of proteins according to the protein abundance, and found that the cost of rhythmic proteins was higher than that of non-rhythmic proteins.

Major comments

1) I am not an expert in proteomics, but according to my knowledge, the proteomics data of mouse liver and cyanobateria used by the authors used SILAC and TMT for protein quantitation respectively, both of which are relatively quantitative methods. Because of the different ionization efficiencies of different peptides, it is not possible to directly compare MS signals of different peptides to determine their abundance in samples, but peaks with the same peptide can be compared in different samples (PMID: 30609196). I checked the data and code using to calculate mouse liver data from Mauvoisin et al., which was used by the author. Although the calculation code was provided, the annotation was not very clear. I think the author used Ratio H/L as the expression of protein abundance. However, Ratio H/L refers to the intensity of each peptide relative to the ratio of the same peptide in the MIX group, which cannot be used for comparison between different proteins. The current relative quantitative method that can be used for comparing proteins can be quantified by label free, processed by MaxQuant, and compared with the IBAQ value therein.

2) The authors calculated the energy expended in protein translation, but not in RNA transcription. The authors assume that the energy cost of RNA transcription is negligible compared to that of protein translation. This is possible in terms of calculating the total energy of transcription and translation, but on the other hand, transcription takes place in the nucleus, and its energy use might be thought of as being in a relatively closed system. Is it possible to calculate the energy of RNA transcription alone? Do the authors think such calculations have biological significance? If agree, I suggest that this work be supplemented.

3) In the absence of other data, the authors have to assume that this noise calculated at the transcriptional level is representative of noise at the protein level, or in any case functionally relevant. I don't think this is a reasonable estimate because the error is too large to make the protein noise calculated using this assumption meaningful. Perhaps the authors should have used other methods to estimate the amount of protein and protein noise from single cell data, for example, ribosome profiling. Or the author can discuss the error of the estimation.

Minor comments

1) To test the hypothesis that genes tend to be rhythmic in the tissues in which they are highly expressed, the authors used a mouse circadian dataset with 11 tissues (transcriptomics from Zhang et al. (2014), Supplementary Table S2). As far as I know, that dataset includes a total of 12 mouse tissues. What about the other tissues? If a selection was made, perhaps the author should state the criteria in the data section.

2) In this paper, gene expression noise is a very important concept. Although the author directly uses the calculation method by Barroso et al. (2018) when quantifying noise expression, I think it is still necessary to explain the quantification process of noise and the biological significance of noise in more detail in the method section.
