# Supplementary material for: Rhythmicity is linked to expression cost at the protein level but to expression precision at the mRNA level
Source: PLoS Comput Biol. 2022 Sep 12;18(9):e1010399. doi: 10.1371/journal.pcbi.1010399 (PMC9518874; doi:10.1371/journal.pcbi.1010399)
Supplement: Attachment — Submitted filename: comments.docx [file pcbi.1010399.s022.docx]

I am glad that the authors have given detailed replies to my previous comments.

In my last review, I mentioned that the abundance of different proteins obtained by previous quantitative methods cannot be compared with each other. The authors reanalyzed it in this revision. It's nice to see that the new results are slightly different from the previous results that there is no change in the conclusions. However, the authors’ reply is not very clear about how to improve the method. I think the authors have moved from analyzing the abundance at the protein level to the peptide level. Is my understanding correct?

As for the second question I mentioned earlier, “whether it is necessary to calculate mRNA-related expression energy”, the authors calculated that the energy required to produce protein was more than 160 times that of mRNA, and the energy cost of protein was much higher than that of mRNA. Therefore, it makes sense to consider just the protein without needing to be very elaborate. I think the explanation given by the author is reasonable.

As for another comment, that transcriptional noise is not representative of translation noise, I would like to thank the authors for providing relevant article on the fact that transcriptional noise is a major source of noise in bacteria. This substitution makes sense in bacteria and yeast. Unfortunately, there is no data on multicellular species, which would make this paper more rigorous.

The author has answered the reasons why the hypothalamus was not included in the analysis. I think the reasons given are reasonable.

In addition, I have a new suggestion for the author to consider.

Major comments

1)As briefly mentioned in the manuscript, other factors such as protein-protein misinteractions, undesirable enzyme activities or antagonistic pleiotropy may also explain the rhythmicity of gene transcription. The authors should provide strong evidences to exclude the possibility that those factors can lead to noise reduction in rhythmic mRNAs.

And no more other comments.
